# Ingestion of Toxic Iron Dose With Benign Outcome

**DOI:** 10.7759/cureus.40103

**Published:** 2023-06-07

**Authors:** Martin J Morales-Cruz, Darielys MejiasMorales, Parker Hunsaker, Erin Butler, Maria Tassone

**Affiliations:** 1 Emergency Medicine, HCA Florida Osceola Hospital, Kissimmee, USA; 2 Emergency Medicine, University of Central Florida College of Medicine, Orlando, USA

**Keywords:** chelation, deferoxamine, toxicology, overdose, iron toxicity

## Abstract

Iron poisoning is a significant and potentially life-threatening condition that is commonly encountered in the emergency department. The severity of toxicity is based on the amount of iron ingested, and symptoms can range from mild gastrointestinal discomfort to multi-organ failure. Although current guidelines recommend therapy for patients with estimated ingestion of >60 mg/kg, the most useful laboratory test to determine toxicity is the serum iron level measured at four to six hours after ingestion. In this report, we present a case of a 28-year-old female who ingested a toxic dose of iron (88 mg/kg) but was only minimally symptomatic and managed with supportive care alone. The case highlights the importance of a high index of suspicion, careful clinical evaluation in patients with iron toxicity, and the need for individualized treatment decisions based on the patient's clinical presentation and laboratory values.

## Introduction

Iron poisoning from intentional or accidental ingestion is one of the most common intoxications and can lead to significant morbidity and mortality. This is due to iron supplements' easy and ready availability in many over-the-counter (OTC) multivitamins. Accidental iron ingestions are more common in children aged five years or younger. In teenagers and adults, it is usually associated with suicide attempts or chronic blood transfusions without adequate monitoring [1].

Failure to diagnose and treat iron poisoning could lead to multi-organ loss and death. However, clinical presentations vary significantly and can overlap with many other ingestions. Therefore, a high index of suspicion, along with a careful history and physical examination, is of utmost importance [2].

Current management guidelines for iron poisoning assess the severity of toxicity at arrival based on clinical presentation and amount ingested and recommend therapy if the level of ingestion is estimated to be >60 mg/kg. However, the most useful laboratory test to determine the patient’s potential for toxicity is the serum iron levels measured four to six hours after ingestion. Peak serum iron levels of over 350 micrograms/dL are associated with significant toxicity [3].

Treatment for iron poisoning depends on the severity of the symptoms and the amount of iron ingested. Deferoxamine, a chelating agent that binds with iron, is the standard treatment for moderate to severe iron toxicity. However, it has potential adverse effects and is costly, making it less practical for patients with minimal symptoms [3]. Therefore, monitoring serum iron levels and clinical symptoms is essential to determine the appropriate management approach. Supportive care, including gastrointestinal decontamination, fluid resuscitation, and close monitoring of vital signs, is the mainstay of treatment for patients with mild to moderate iron toxicity.

This report aims to increase awareness of iron poisoning by providing an evidence-based approach to diagnosing and managing this condition by presenting a case of a young woman who was minimally symptomatic despite ingesting a toxic dose of iron. We highlight the importance of considering iron poisoning in patients presenting with unexplained symptoms and the need for timely diagnosis and appropriate treatment. Additionally, by reviewing the current literature on iron toxicity, we provide insights into the epidemiology, clinical presentation, diagnosis, and management of this potentially fatal condition.

## Case presentation

A 28-year-old female with no significant past medical history presented to the emergency department via ambulance two hours after an intentional overdose of iron and magnesium oxide. The patient reported oral ingestion of approximately 90 tablets of ferrous sulfate 65 mg and 20 capsules of magnesium oxide 400 mg. Based on the reported ingestion and the patient's weight (67.8 kg), elemental iron consumption was 88 mg/kg, considered toxic levels. On initial evaluation, the patient was complaining of abdominal discomfort and nausea. Shortly after arrival, she experienced two episodes of bilious emesis. The patient was afebrile and had a pulse of 85 beats per minute, respiratory rate of 18 breaths per minute, blood pressure of 122/73 mmHg, and oxygen saturation of 97% on room air. Physical examination was only remarkable for mild tenderness to palpation of the mid-abdomen. Intravenous fluids and antiemetics were provided. The poison control center was contacted and the specialist recommended an abdominal radiograph, and iron levels at four and six hours after ingestion. Since the patient ingested the abovementioned supplements as part of a suicide attempt, she was also tested for other common co-ingestions, and psychiatry was consulted. The poison control specialist recommended against charcoal or deferoxamine due to the lack of symptoms. They recommended deferoxamine only if the patient became unstable and whole bowel irrigation if radiopaque tablets were seen on imaging. The initial workup revealed a normal complete blood count, normal electrolytes, negative acetaminophen, salicylate, and ethanol levels, and a negative pregnancy test.

Electrocardiogram revealed a normal sinus rhythm with a ventricular rate of 85 beats per minute. Chest and abdominal radiographs were unremarkable, and no tablets were visualized. The patient was admitted to the intensive care unit. Iron levels were 344 ug/dL at four hours and 266 ug/dL at six hours. Consideration was given to administering deferoxamine, but chelation therapy was withheld since the patient remained stable and asymptomatic. The decision was discussed with the Poison Control Center, and they agreed with supportive management and close monitoring. The patient was discharged home after her psychiatric clearance on admission day three.

## Discussion

Iron intoxication is commonly seen in the emergency department. The 2015 Annual Report of the American Association of Poison Control Centers (AAPCC) National Poison Data System reported 4072 exposures to iron or iron salts, of which 21% were intentional ingestion as part of a suicide attempt and 50% were unintentional ingestions by children aged five years or younger [4]. Young children are particularly at risk of accidental ingestion because of their increased level of curiosity and oral phase of development. Furthermore, some iron tablets have an appearance similar to chocolate candy.

Rapid diagnosis and intervention are key during an initial encounter with an intoxicated patient in the emergency department; therefore, toxicological screening provides limited value, and one should rely on a detailed clinical evaluation. Patients with iron toxicity could present with a spectrum of symptoms, from mild gastrointestinal discomfort to severe tissue damage and organ failure [1]. Current guidelines divide iron toxicity into five stages based on the time from ingestion and presenting symptoms. The first stage is from 0.5 to six hours, presenting gastrointestinal symptoms such as abdominal pain, vomiting, diarrhea, hematemesis, and hematochezia. The second stage is an apparent recovery phase at six to 24 hours. The third stage is the recurrence of gastrointestinal symptoms, metabolic acidosis, possible shock, or organ failure (six to 72 hours). The fourth stage is 12 to 96 hours, characterized by elevation of aminotransferase levels/hepatic failure. Lastly, the fifth stage represents long-term changes seen two to eight weeks after ingestion, presenting gastric scarring and liver fibrosis [5,6]. The usefulness of this staging method is questionable since not every patient goes through every stage, and many patients progress rapidly compared to others. Therefore, one should rely more on clinical manifestations rather than the time of ingestion.

Peak serum iron levels are achieved two to six hours after ingestion. Newer formulations include sustained-release or enteric-coated pills, for which serial monitoring of iron levels for eight to 10 hours or until down-trending is necessary. Peak serum iron levels above 350 micrograms/dL or 20-60 mg/kg have been associated with toxicity [5,7,8]. Iron is rapidly cleared from the serum and deposited in the liver. As the body content of iron increases and plasma iron-binding protein transferrin becomes saturated, iron starts binding to other proteins and molecules. Therefore, iron levels measured after its peak may need to be more accurate, misleading the management plan. Also, as seen in our case, it is important to consider co-ingestions. The fact that our patient co-ingested magnesium oxide possibly helped decrease the absorption and toxicity of the ingested iron since magnesium oxide is known to affect the absorption of many medications/vitamins when taken in conjunction.

In addition to iron serum levels, one should consider other causes of the patient’s symptoms and evaluate for possible effects of the intoxication. Additional workups may include leukocyte count, electrolytes, serum glucose, coagulation studies, lipase, and hepatic enzymes. Plain radiographs may reveal radiopaque iron tablets in the gastrointestinal tract about two to six hours after ingestion, but normal radiographs do not exclude iron ingestion, as our patient’s presentation demonstrates. Also, the number of tablets seen on the radiograph does not correlate with the severity of intoxication; instead, poisoning is closely linked to the concentration of elemental iron [9]. Significant vomiting or diarrhea, shock, coma, iron tablets on abdominal radiographs, coagulopathy, metabolic acidosis, hyperglycemia, and leukocytosis all correlate with elevated serum iron [5,6].

There is conflicting evidence in terms of the treatment of iron poisoning. Current guidelines recommend therapy determination based on clinical presentation, the amount ingested, and serum iron levels. However, there are several limitations: clinical manifestations vary widely, the amount ingested is rarely accurately reported by patients, and serum iron levels can take hours to days to come back, depending on the institution. Deferoxamine chelation therapy is the treatment of choice for iron poisoning. Gastric lavage is also indicated if >20 mg/kg of iron has been ingested. Whole bowel irrigation is indicated for those whose abdominal X-rays reveal iron tablets beyond the pylorus or whose serum iron level continues to rise despite decontamination efforts [8]. Activated charcoal binds iron poorly and is not practical.

More recent studies favor supportive management for patients who present only minimally symptomatic, as the patient discussed in this report. Deferoxamine should be held until the iron level is 500 mg/dl due to serious side effects of deferoxamine, especially when provided as an infusion for >24 hours to prevent hypotensive shock and acute lung injury [3]. It is important to note that no absolute can be made just with a single level. Patients can be safely discharged after asymptomatic observation between six and 12 hours, iron levels below 350 mg/dl and down trending, and a normal anion gap.

## Conclusions

Iron poisoning can be challenging since patients usually present with non-specific symptoms, provide unreliable history due to intoxication, and frequently co-ingest other substances. Peak serum iron levels are achieved two to six hours after ingestion. Therefore, initial management should be based on clinical presentation, requiring high clinical suspicion. Current guidelines recommend chelation therapy based on peak serum iron levels. However, as discussed in this report, patients can have toxic levels of iron without having significant symptoms of intoxication. Consequently, one should determine the risks versus benefits of chelation therapy for minimally symptomatic patients and instead consider supportive management along with close monitoring of clinical progression in the intensive care unit.
